# How Not To Fix a Tibial Fracture: A Case Report on Treatment By a Quack

**DOI:** 10.7759/cureus.40203

**Published:** 2023-06-09

**Authors:** Roop Singh, Isha Seth, Aditya Seth, Sunayana Singh, Ram K Aiyappan, Chander Mohan Yadav, Harsh Jain, Anish Tawde, Gaurav K Agrawal, Aditi jain

**Affiliations:** 1 Orthopaedics, Pandit Bhagwat Dayal Sharma Post Graduate Institute of Medical Sciences, Rohtak, IND; 2 Obstetrics and Gynaecology, Amrita Hospital, Faridabad, IND; 3 Orthopaedics, Krishna Institute of Medical Sciences (KIMS) Sunshine Hospital, Hyderabad, IND; 4 Obstetrics and Gynecology, Government Medical College and Hospital, Chandigarh, IND; 5 General Surgery, Amrita Hospital, Faridabad, IND; 6 Orthopaedics and Rehabilitation, Pandit Bhagwat Dayal Sharma Post Graduate Institute of Medical Sciences, Rohtak, IND; 7 Arthroplasty, Krishna Institute of Medical Sciences (KIMS) Sunshine Hospital, Hyderabad, IND; 8 Internal Medicine, Sawai Man Singh Medical College, Jaipur, IND

**Keywords:** mismanagement, infections, non-union, tbs (traditional bone setter), quack

## Abstract

Quackery in the orthopaedic profession has existed for quite a long time. Due to the shortage of orthopaedic healthcare staff in public hospitals and the high costs in private facilities, members of disadvantaged communities turn to unlicensed and unskilled practitioners (quacks). The main factors responsible for the increased number of quacks performing orthopaedic treatment are illiteracy, high treatment cost, mismatch in the orthopaedic surgeon-to-population ratio, especially in rural areas, and the absence of any form of health insurance. Moreover, their easy availability and offer of low-cost treatment draw innocent and illiterate patients to them, even though these quacks perform orthopaedic treatment in the most unhygienic, unsterilized, and unconventional manner. The government should intervene and take measures to make orthopaedic treatment more affordable and accessible, especially to the rural population.

## Introduction

A quack is an individual who presents themselves as having professional competence or credentials they don't actually possess, often in the field of medicine. Quackery involves promoting false or unproven medical practices, including deceitful representation of one's abilities and experience in diagnosing and treating illnesses and false claims about the results of the treatments offered [1]. Most individuals engaging in quackery lack formal education or professional training. They have acquired knowledge either through family tradition, where it has been passed down as a family business, or through their work as assistants (such as nurses, X-ray technicians, plaster technicians, operation theater assistants, etc.) in orthopaedic departments, where they have observed orthopedic surgeons performing professional treatments. They then start their own bone-setting business, especially in rural and semi-urban areas, and start doing low-cost specialized orthopaedic treatment by using unconventional, unsterilized, and old instruments available in the market leading to undesirable and harmful effects on patients' health.

In developing nations like India, many fractures are treated by Traditional Bonesetters (TBS), especially in rural areas. Despite high patronage, TBS remains an untrained quack whose practice is often associated with many complications and high morbidity [2,3]. These quacks often make questionable or ineffective diagnoses and perform pseudo-management of orthopaedic fractures and injuries. They also perform injudicious massage and herbal oil application in case of soft tissue injuries, while traction and splinting are the only treatment given in case of fractures. However, such treatment often leads to myositis ossificans, infection, deformities, non-union, malunion, or gangrene in case of vascular injury [4].

These complications are often seen in children, especially when parents are told that surgery is the only option to manage injury or deformity. Often, such parents would do anything to avoid surgeries on their wards and would end up visiting the quacks that promise a quick and easy fix. Unfortunately, patients who fall prey to such practices often end up with a lifetime of disability, as they rarely escape such complications [4].

We report a case of a 52-year-old male who presented to the emergency department after a fall. The patient had received treatment for a tibial fracture one year earlier by a Traditional Bonesetter (TBS) with screw fixation, which subsequently became infected, resulting in exposure of the bone and implant. This case report aims to demonstrate the extent of illiteracy in rural India and the level of trust placed by its people in fraudulent activities. All investigations and reporting of this case were conducted in accordance with the institution's ethical research principles, and informed consent was obtained from the patient prior to participation in the study.

## Case presentation

A 52-year-old male laborer presented to the Accident and Emergency Department with a history of slip and fall two days back, with pain in his right leg. The patient reported a prior history of a roadside accident one year back, resulting in a closed fracture of his right tibia and fibula. The patient was treated by a well-known Traditional Bonesetter (TBS) in his village, who promised that his treatment would result in fracture union and early mobility at a minimal cost. The TBS, described by the patient was an older adult in his 60s, who had inherited the secret art of setting broken bones from his forefathers who had also practiced traditional bone setting.

After administering local anesthesia to the patient around the fracture site, the TBS inserted screws without using a drill bit with the help of a screwdriver and without obtaining any radiographs or conducting any investigations. The wound was then closed without maintaining an aseptic environment in a small room at the TBS's workplace. The dressing was applied, and the patient was discharged the next day with a prescription for painkillers and herbal medicine, and he was advised against weight-bearing for the next 14 days. Within the first month, the patient developed erythema and discharge from the incision site, and the TBS provided regular dressing changes. After one month, the patient was able to walk with a cane, but the bone remained exposed, and there was still discharge from the incision site. The patient was told that the discharge would stop within a few months and was given a repeat prescription for herbal medicine. However, over the following months, although the amount of discharge reduced, a significant amount of bone with a few screws remained exposed. Despite this, the patient continued to trust the TBS and did not seek medical care from any government or private hospital.

**Figure 1:**
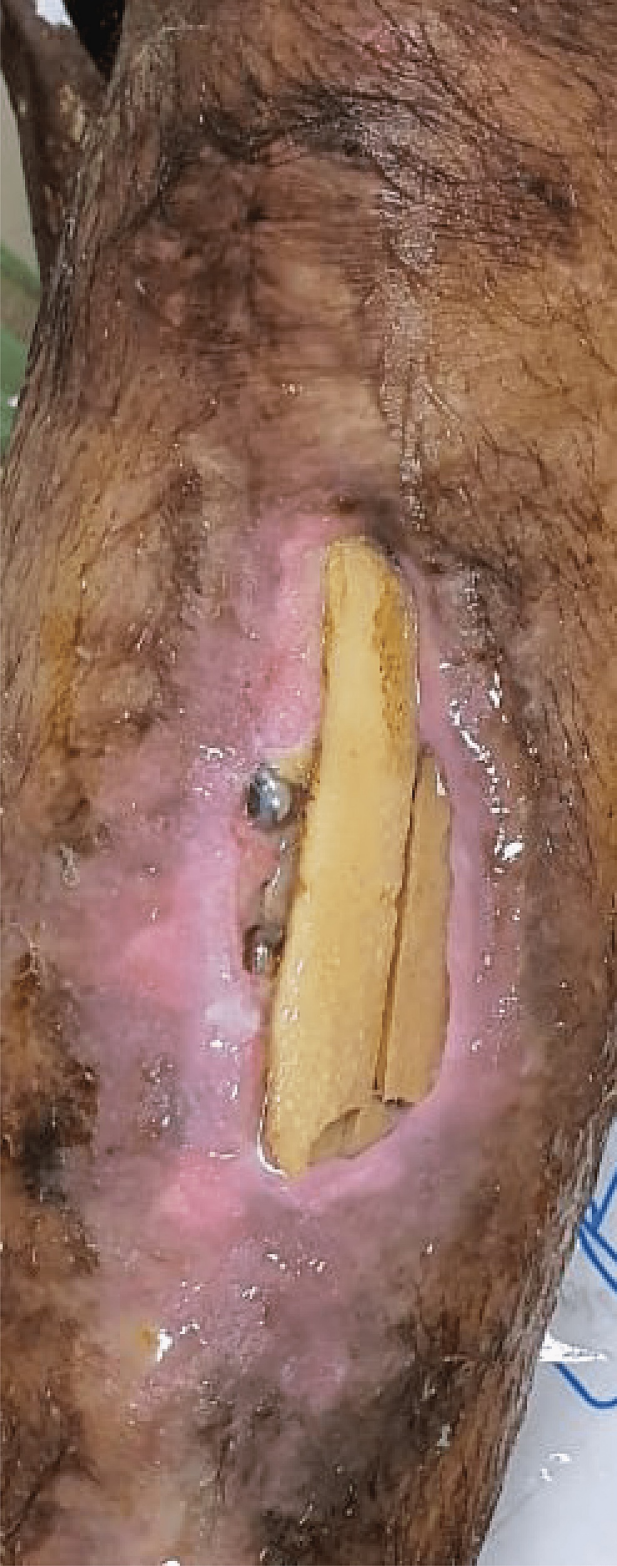
Wound of the patient upon arrival Erythematous skin with dead necrotic bone with exposed screws can be seen on antero-lateral surface of the tibia. There is also a fracture of the exposed bone.

**Figure 2:**
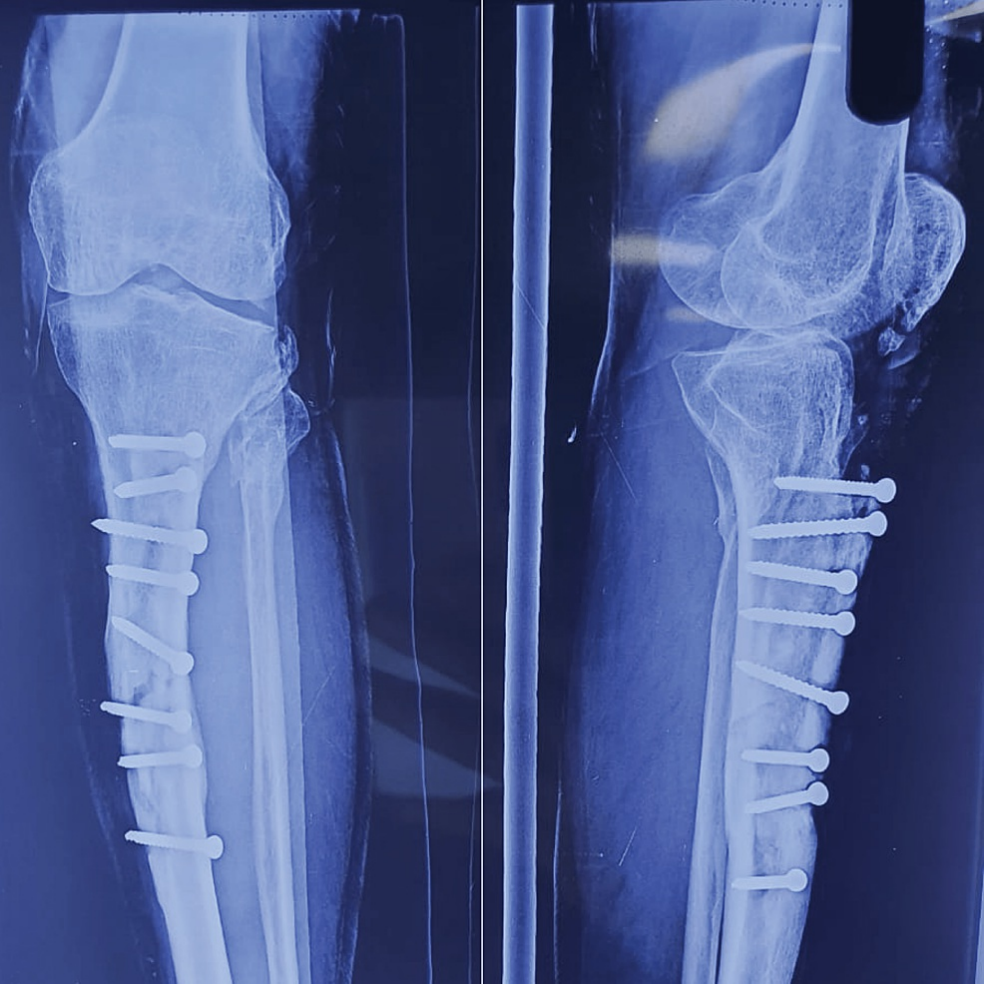
X-ray image of the patient's right leg on presentation

After one year of receiving treatment from the TBS, the patient had a slip and fall accident. However, still unwilling to go to a hospital, the patient returned to the same TBS who was unavailable at his workplace. After waiting for two days, the patient eventually presented at our tertiary care center with an inability to bear weight, poor skin condition, exposed dead bone, and a fracture due to the fall (Figure 1). A plain radiograph of the leg revealed multiple screws in the tibia with local osteolysis (Figure 2). Routine blood samples were taken, revealing a raised TLC count of 13,400/mm^3^, an erythrocyte sedimentation rate (ESR) of 52 mm/hr, and a C-reactive protein (CRP) level of 21 mg/L.

**Figure 3:**
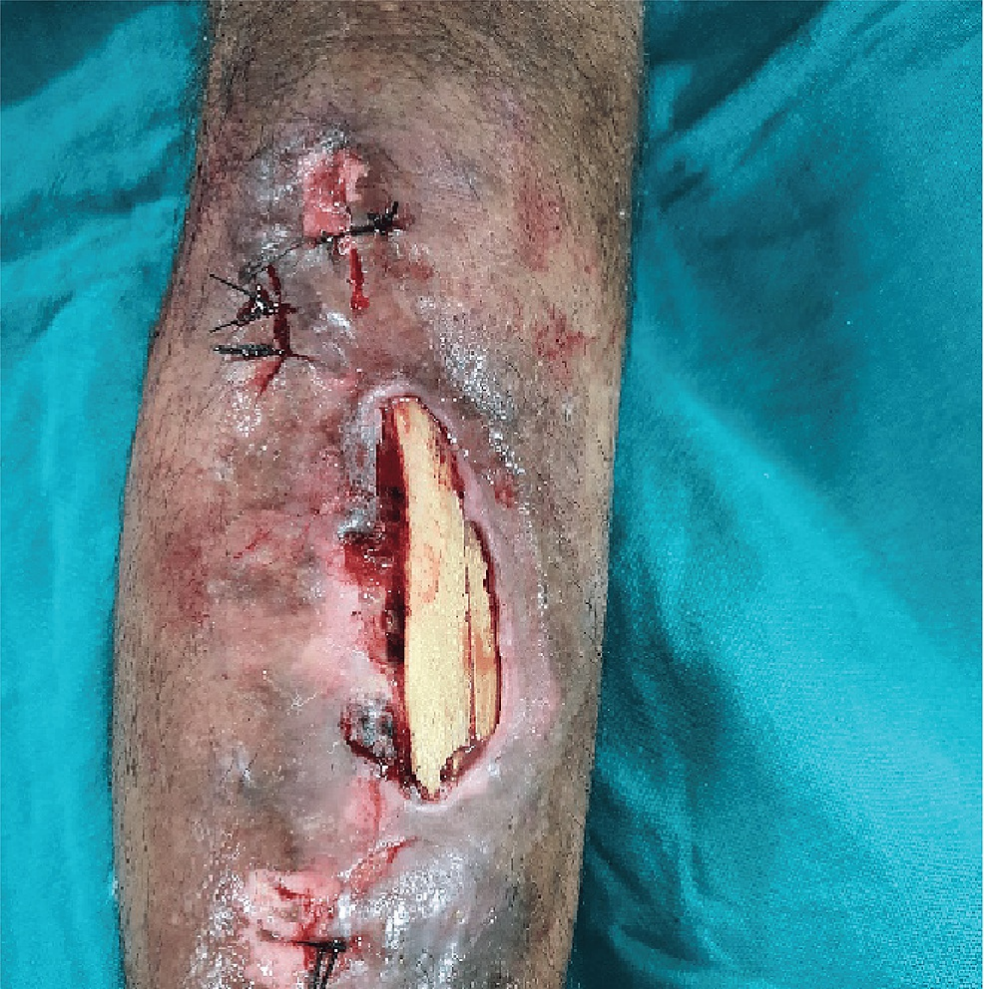
Intra-operative images after removal of screws under fluoroscopic guidance

**Figure 4:**
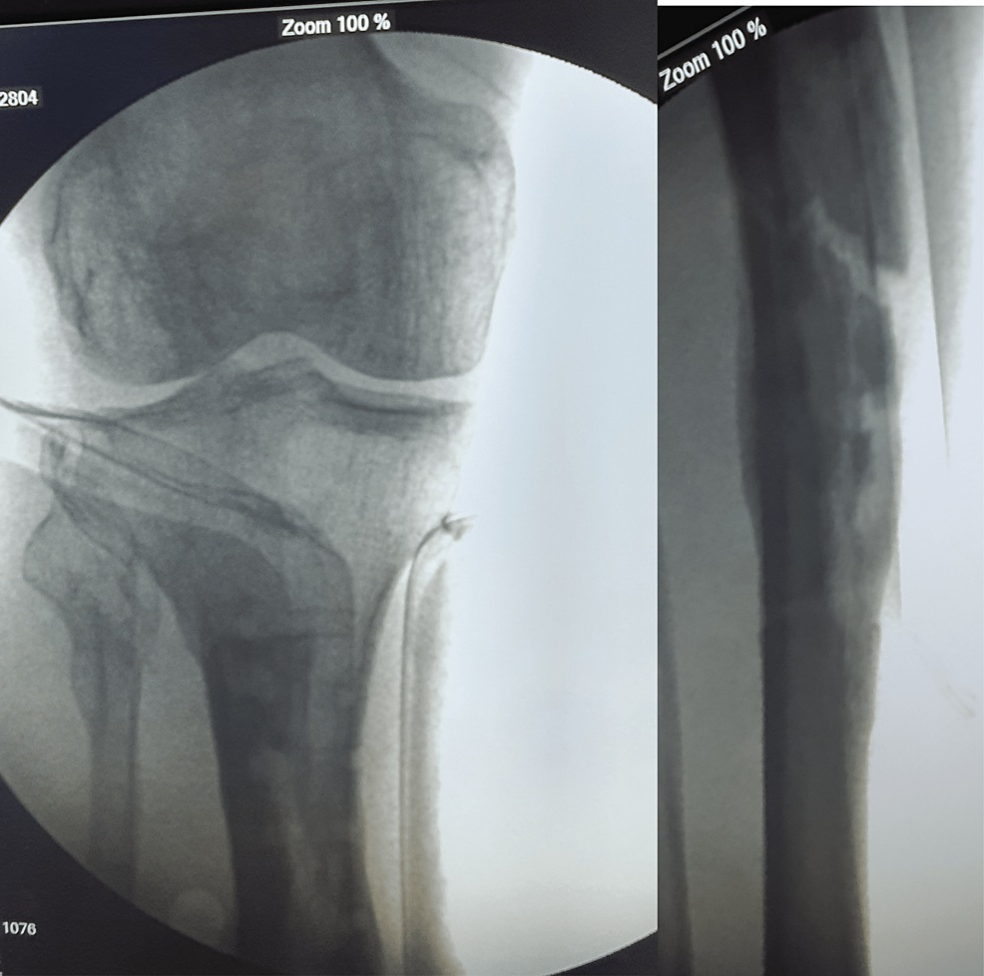
Fluoroscopic images after screw removal.

The patient was admitted, and broad-spectrum antibiotics were started. A staged surgical management plan was made in consultation with the plastic surgeon. In stage I, wound debridement was performed, and the excised material was sent for pus culture and biopsy. The loose infected screws were removed under fluoroscopic guidance (Figures 3-4). Stage II of the management plan involved the removal of dead bone, flap coverage of the wound, and stabilization after control of local infection and biopsy report. However, after two days, the patient left against medical advice. When asked why he was leaving the hospital and where he would get treatment, the patient replied that he had a family to look after in his village and that the TBS was available at his centre now.

## Discussion

It is in the most economically backward countries that the art of traditional bone setting is still practiced [2,3,5,6]. Such unethical and fraudulent activity, performed by people with no medical background, often results in enormous complications, which can be as devastating as Volkmann ischaemic contracture or compartment syndrome, resulting in limb amputation. This is often due to the application of tight splints and bandages for the treatment of simple fractures, sprains, or painful joints [7-9].

Our patient preferred treatment from a TBS due to his belief in old methods, unavailability of affordable medical facilities in his area, lack of belief in allopathic medicine, and to avoid the loss of income that might occur by traveling to and getting treated at a secondary or tertiary care health center. A fracture that could have been treated by a simple intramedullary nail ended up being an open fracture with exposed dead bone and osteomyelitis. Such devastating complications could have been avoided if the patient had not visited the quack for treatment. Nevertheless, despite these complications, our patient still believed that the quack had his best interest in mind and offered much better and affordable treatment.

In India, it is estimated that there are roughly 70,000 traditional bonesetters who manage approximately 60 percent of all bone trauma cases [10]. Most of these quacks operate in remote communities where there are no basic medical services. This is the reality in many other emerging nations [11].

Within the existing literature, multiple bone-setting approaches have been documented, demonstrating comparable or superior outcomes when compared to conventional practices [10]. Hemmila et al. [12] conducted a randomized clinical trial, employing bone-setting techniques, which yielded superior results in treating chronic back pain compared to standard physiotherapy sessions. In the context of adult forearm bone fractures, open reduction, and plate fixation have been widely accepted as the standard treatment. However, Shang et al. [13] investigated a Chinese method involving bone separator pads and splint immobilization in a substantial sample of 2,221 forearm fracture cases. Their findings not only showcased the simplicity, cost-effectiveness, and effectiveness of this approach but also highlighted its ability to eliminate delayed union or non-union. Similarly, Fang et al. [14] explored the utilization of paper roll spreaders and wooden splints in a cohort of 147 patients with forearm fractures. Their observations indicated that by preserving the interosseous membrane, the manipulative reduction process was significantly simplified. Furthermore, they found that simple wooden splints surpassed the plaster of Paris in terms of efficacy and patient satisfaction when immobilizing fractures in the shafts of both forearm bones. In addition, randomized trials focusing on buckle fractures of the distal radius revealed successful treatment outcomes through the use of soft bandages [15]. This technique offers advantages such as simplicity, cost-effectiveness, and enhanced comfort, particularly for pediatric patients. Notably, the contemporary practice known as 'functional cast bracing,' advocated by Sarmiento and Latta [16], exhibits striking similarities to certain bandaging patterns employed by traditional bone healers, reminiscent of their use of 'bamboo' bandaging techniques.

Despite the existence of successful bone-setting procedures, it is important to acknowledge that the practice has also encountered significant shortcomings and criticism [10]. Providers of bonesetting have faced widespread criticism for their utilization of methods deemed 'irrational' by contemporary medical standards. Oginni LM [17] conducted a study and found a notably high failure rate of 66.7% among patients who voluntarily opted out of traditional bone-setting treatment. The traditional bandaging technique, involving the direct application of splints to the skin, has often been ridiculed as a mere variation of the outdated tourniquet fracture splint [18]. Moreover, the literature contains numerous accounts of severe complications arising from bone-setting practices [18-21], painting a grim picture of the potential risks associated with such procedures.

Strict laws and regulations, education programs for the general public, and availability of adequate medical facilities even in remote locations can rid people of complications of traditional bone-setting. Nevertheless, until affordable healthcare facilities reach the grassroots level of society, great short-term benefits, in terms of minimizing the complications of traditional bone setting, can be derived from imparting primary health education and training in orthopedic care to traditional bonesetters [22]. In Ethiopia, Eshete M reported a reduction in amputation rates after a one-day instructional course offered to traditional bonesetters [23]. Similar optimistic results were derived by Shah et al. [24] and Onuminya JE [2] in their respective nations.

## Conclusions

To conclude, this case is presented to highlight the need of the hour, that the government of India and the state governments should strengthen basic level healthcare facilities in rural areas, organize campaigns to educate people and TBS to minimize these complications, and at the same time, punish these unscrupulous TBS through legislation. Our Indian orthopaedic association, especially the one working in rural society, can play a significant role in decreasing such types of complications by organizing awareness campaigns and treatment camps regularly throughout the country.
